# Development of a predictive model for 1-year postoperative recovery in patients with lumbar disk herniation based on deep learning and machine learning

**DOI:** 10.3389/fneur.2024.1255780

**Published:** 2024-06-11

**Authors:** Yan Chen, Fabin Lin, Kaifeng Wang, Feng Chen, Ruxian Wang, Minyun Lai, Chunmei Chen, Rui Wang

**Affiliations:** ^1^Pingtan Comprehensive Experimentation Area Hospital, Pingtan, China; ^2^Fujian Medical University Union Hospital, Fuzhou, Fujian, China; ^3^Fujian Medical University, Fuzhou, Fujian, China

**Keywords:** predictive model, machine learning, deep learning, lumbar disk herniation, lumbar JOA score

## Abstract

**Background:**

The aim of this study is to develop a predictive model utilizing deep learning and machine learning techniques that will inform clinical decision-making by predicting the 1-year postoperative recovery of patients with lumbar disk herniation.

**Methods:**

The clinical data of 470 inpatients who underwent tubular microdiscectomy (TMD) between January 2018 and January 2021 were retrospectively analyzed as variables. The dataset was randomly divided into a training set (*n* = 329) and a test set (*n* = 141) using a 10-fold cross-validation technique. Various deep learning and machine learning algorithms including Random Forests, Extreme Gradient Boosting, Support Vector Machines, Extra Trees, K-Nearest Neighbors, Logistic Regression, Light Gradient Boosting Machine, and MLP (Artificial Neural Networks) were employed to develop predictive models for the recovery of patients with lumbar disk herniation 1 year after surgery. The cure rate score of lumbar JOA score 1 year after TMD was used as an outcome indicator. The primary evaluation metric was the area under the receiver operating characteristic curve (AUC), with additional measures including decision curve analysis (DCA), accuracy, sensitivity, specificity, and others.

**Results:**

The heat map of the correlation matrix revealed low inter-feature correlation. The predictive model employing both machine learning and deep learning algorithms was constructed using 15 variables after feature engineering. Among the eight algorithms utilized, the MLP algorithm demonstrated the best performance.

**Conclusion:**

Our study findings demonstrate that the MLP algorithm provides superior predictive performance for the recovery of patients with lumbar disk herniation 1 year after surgery.

## Introduction

Lumbar disk herniation (LDH) is a common and frequently occurring disease that is the most common cause of back and leg pain, resulting in great suffering such as reduced ability to work and learn, reduced quality of life, and even disability (1). Surgery, especially tubular microscopic discectomy (TMD), has become the conventional treatment for LDH in recent years (2). TMD is a minimally invasive method to remove the herniated disk from the posterior approach using surgical microscopic instruments. However, there are several factors that can affect postoperative recovery (3). Clinical predictive modeling (CPM) is a statistical model based on multiple pathologies of the disease that can predict the risk of certain future outcomes in patients with certain characteristics (4, 5). Building statistical models requires a large amount of clinical data, and machine learning (ML) algorithms can accurately process the raw data, analyze the connections between important data, and make accurate decisions (6). With the widespread use of machine learning, deep learning, as an important branch of machine learning, has advantages in automatic feature learning and function simulation construction (7–9). Due to the complexity and size of clinical data, using deep learning models and machine learning can improve the accuracy of models and predictions in data processing, as well as in building clinical models (10, 11). The goal of this study is to develop a predictive model based on deep learning and machine learning for the recovery of patients with lumbar disk herniation 1 year after surgery.

## Methods

All data for this study were obtained from the Department of Neurosurgery, Fujian Medical University Union Hospital. The study recorded the medical variables of patients who were hospitalized and underwent TMD between January 2016 and January 2018. The data included patients’ basic information, medical history, physical examination, preoperative test results, and preoperative scores. Retrospective analysis was conducted, and deep learning and machine learning algorithms were used to establish a predictive model for the 1-year postoperative recovery of patients with lumbar disk herniation.

### Inclusion criteria

(1) Age of inclusion: 12–85 years old; (2) The prominent lumbar segments are: L3/4, L4/5, or L5/S1, including cases of combined protrusions involving two or three segments. (3) have typical sciatica with or without lumbar pain and other symptoms; (4) those who have been ineffective after standardized conservative treatment for more than 3 months and seriously affect their lives, or those with severe pain, cauda equina dysfunction, muscle strength loss, muscle atrophy, and other symptoms; (5) the straight leg raising test on the affected side is less than or equal to 70°; (6) confirmed by CT and MRI lumbar disk protrusion, and the location of the protrusion matches the corresponding neurological symptoms; and (7) receiving standardized unilateral paraspinal tubular microdiscectomy (TMD) technology treatment and a consistent physical therapy regimen (12, 13).

For more information about this study and the standardized surgical procedures at our institution, please refer to our previously published study (14).

### Exclusion criteria

(1) Those with missing imaging data or unable to follow up as required; (2) those with segmental lumbar instability suggested by frontal and lateral lumbar X-ray and hyperextension and hyperflexion; (3) those with other serious physical, psychological, or mental diseases; (4) those with rheumatic immune diseases that may cause similar symptoms; and (5) those who are participating in other clinical trials.

### Data collection

To construct and validate the prognostic model, we retrospectively collected clinical data related to patients with LDH who met the inclusion and exclusion criteria. The potential predictors included 42 variables related to patients’ medical history, examination, and preoperative test results, with the cure rate of the lumbar Japanese Orthopedic Association (JOA) score 1 year after TMD as the outcome measure.

The following variables were included as factors in the analysis: age, gender, height, weight, body mass index (BMI), high-risk occupation (occupations that require prolonged sedentary or high-intensity physical activity), family history (with first-degree relatives affected by LDH), history of lumbar trauma, duration of disease, duration of preoperative conservative treatment, duration of preoperative pain medication, low back pain, underlying diseases (hypertension, diabetes), history of smoking, history of alcohol abuse, angle of preoperative physical examination (as measured by the straight leg raise test), sensory impairment, muscle strength classification of the affected limb, Barthel scale, serum creatine kinase (CK), and lumbar degeneration, associated lumbar disk herniation, American Society of Anesthesiologists (ASA) grading, Oswestry Disability Index (ODI) score, preoperative low back pain and leg pain numerical rating scale (NRS) scores, the number of surgical segments as determined by the JOA, surgical time, and intraoperative bleeding. These are shown in Table 1. The cure rate score of the lumbar JOA 1 year after TMD surgery was also used as an outcome measure. Further details on these factors are provided in Supplementary material 1.

**Table 1:** Descriptive statistics of different influencing factors in a study population grouped by whether the improvement in lumbar JOA score was >60% 1 year after TMD.

Variables	Total (470)	JOA ≤ 60% (271)	JOA > 60% (199)	*p*-value
Age	51.81 ± 14.36	54.31 ± 14.02	48.40 ± 14.14	<0.001
Preop_JOA	13.11 ± 3.43	12.75 ± 3.09	13.59 ± 3.80	<0.001
Preop_ODI	48.29 ± 20.24	50.66 ± 19.00	45.05 ± 21.43	<0.001
Intraop bleeding	22.73 ± 13.56	24.23 ± 15.84	20.68 ± 9.28	<0.001
Surgery time	2.89 ± 0.75	2.99 ± 0.72	2.76 ± 0.77	<0.001
SD	1.37 ± 0.19	1.38 ± 0.16	1.35 ± 0.22	0.2883
Gender				0.5189
Female	208(44.26)	116(42.80)	92(46.23)	
Male	262(55.74)	155(57.20)	107(53.77)	
Height				<0.001
<40	102(21.70)	68(25.09)	34(17.09)	
40 ~ <60	159(33.83)	72(26.57)	87(43.72)	
≥60	209(44.47)	131(48.34)	78(39.20)	
Weight				0.1359
<50	39(8.30)	23(8.49)	16(8.04)	
<60	131(27.87)	62(22.88)	69(34.67)	
<70	180(38.30)	113(41.70)	67(33.67)	
<80	82(17.45)	49(18.08)	33(16.58)	
<90	26(5.53)	16(5.90)	10(5.03)	
≥90	12(2.55)	8(2.95)	4(2.01)	
BMI				<0.001
<18.5	7(1.49)	3(1.11)	4(2.01)	
<24	193(41.06)	90(33.21)	103(51.76)	
<28	228(48.51)	153(56.46)	75(37.69)	
≥28	42(8.94)	25(9.23)	17(8.54)	
History of lower back trauma				1.0000
No	452(96.17)	261(96.31)	191(95.98)	
Yes	18(3.83)	10(3.69)	8(4.02)	
Hypertension				0.1546
No	374(79.57)	209(77.12)	165(82.91)	
Yes	96(20.43)	62(22.88)	34(17.09)	
Diabetes				0.0838
No	428(91.06)	241(88.93)	187(93.97)	
Yes	42(8.94)	30(11.07)	12(6.03)	
Alcohol use				<0.001
No	406(86.38)	221(81.55)	185(92.96)	
Yes	64(13.62)	50(18.45)	14(7.04)	
Smoking				0.3361
No	361(76.81)	213(78.60)	148(74.37)	
Yes	109(23.19)	58(21.40)	51(25.63)		Variables	Total (470)	JOA ≤ 60% (271)	JOA > 60% (199)	*p*-value
ASA				0.3566
1	305(64.68)	171(62.73)	134(67.34)	
2	152(32.34)	90(33.21)	62(31.16)	
3	13(2.77)	10(3.69)	3(1.51)	
Family history				0.2410
No	419(89.15)	246(90.77)	173(86.93)	
Yes	51(10.85)	25(9.23)	26(13.07)	
Preop_Painkiller				0.0014
No	295(62.77)	153(56.46)	142(71.36)	
Yes	175(37.23)	118(43.54)	57(28.64)	
Preop_Hormone				0.4476
No	438(93.19)	250(92.25)	188(94.47)	
Yes	32(6.81)	21(7.75)	11(5.53)	
CTT				0.1501
≤6	290(61.70)	162(59.77)	104(64.32)	
≤12	43(9.15)	22(8.12)	21(10.55)	
≤24	75(15.96)	53(19.56)	22(11.06)	
>24	62(13.19)	34(12.55)	28(14.07)	
WLPT				0.0053
≤6	201(42.98)	112(41.32)	89(45.23)	
≤12	76(15.96)	43(15.87)	33(16.08)	
≤24	43(9.15)	15(5.54)	28(14.07)	
>24	150(31.91)	101(37.27)	49(24.62)	
Lumbago				0.0587
No	113(24.04)	56(20.66)	57(28.64)	
Yes	357(75.96)	215(79.34)	142(71.36)	
SLETA				0.6709
<40	157(33.40)	93(34.32)	64(32.16)	
40–<60	202(42.98)	118(43.54)	84(42.21)	
≥60	111(23.62)	60(22.14)	51(25.63)	
DOS				0.2122
Nothing	307(65.32)	181(66.79)	126(63.32)	
Mild	137(29.15)	72(26.57)	65(32.66)	
Obvious	26(5.53)	18(6.64)	8(4.02)	
MS				0.4210
1	2(0.43)	2(0.74)	0	
2	1(0.21)	1(0.37)	0	
3	5(1.06)	4(1.48)	1(0.50)	
4	91(19.15)	54(19.93)	37(18.09)	
5	371(78.94)	210(77.49)	161(80.90)	
Babinski				0.9225
Negative	459(97.66)	264(97.42)	195(97.99)	
Positive	11(2.34)	7(2.58)	4(2.01)	
CK				0.4958
≤198	431(91.70)	246(90.77)	185(92.96)	
>198	39(8.30)	25(9.23)	14(7.04)	
Number				<0.001
1	129(27.45)	44(16.24)	85(42.71)	
2	292(62.13)	199(73.43)	93(46.73)	
3	29(6.17)	14(5.17)	15(7.54)	
4	16(3.40)	12(4.43)	4(2.01)	
5	4(0.85)	2(0.74)	2(1.01)	
SSN				0.2220
1	460(97.87)	263(97.05)	197(98.99)	
2	8(1.70)	7(2.58)	1(0.50)	
3	2(0.43)	1(0.37)	1(0.50)	
Protrusion direction				<0.001
Left	346(73.62)	224(82.66)	122(61.31)	
Right	124(26.38)	47(17.34)	77(38.69)	
Collapse				0.7114
No	392(83.40)	228(84.13)	164(82.41)	
Yes	78(16.60)	43(15.87)	35(17.59)	
LS				1.0000
No	450(95.74)	259(95.57)	191(95.98)	
Yes	20(4.26)	12(4.43)	8(4.02)	
Osteoporosis				0.0284
No	424(90.21)	237(87.45)	187(93.97)	
Yes	46(9.79)	34(12.55)	12(6.03)	
Calcification				<0.001
No	126(26.81)	44(16.24)	82(41.21)	
Yes	344(73.19)	227(83.76)	117(58.79)	
Sagittal_Disc_Herniation_Pos				0.0053
−3	3(0.64)	0	3(1.51)	
−2	21(4.47)	8(2.95)	13(6.53)	
−1	313(66.60)	198(73.06)	115(57.79)	
0	117(24.89)	59(21.77)	58(29.15)	
1	13(2.77)	5(1.85)	8(4.02)	
2	3(0.64)	1(0.37)	2(1.01)	
Location				<0.001
1	117(24.89)	52(19.19)	65(32.66)	
2	305(64.89)	201(74.17)	104(52.26)	
3	44(9.36)	17(6.27)	27(13.57)	
4	4(0.85)	1(0.37)	3(1.51)		Variables	Total (470)	JOA ≤ 60% (271)	JOA > 60% (199)	*p*-value
Grade				<0.001
1	101(21.49)	41(15.13)	60(30.15)	
2	309(65.74)	201(74.17)	108(54.27)	
3	60(12.77)	29(10.70)	31(15.58)	
Modic change				<0.001
0	105(22.34)	39(14.39)	66(33.17)	
1	244(51.91)	176(64.94)	68(34.17)	
2	55(11.70)	26(9.59)	29(14.57)	
3	66(14.04)	30(11.07)	36(18.09)	
Pfirrmann				<0.001
1	2(0.43)	0	2(1.01)	
2	29(6.17)	10(3.69)	19(9.55)	
3	102(21.70)	32(11.81)	70(35.18)	
4	286(60.85)	191(70.48)	95(47.74)	
5	51(10.85)	38(14.02)	13(6.53)	
Lumbago_NRS				<0.001
0–2	104(22.13)	35(12.92)	69(34.67)	
3–4	241(51.28)	166(61.25)	75(37.69)	
5–6	90(19.15)	52(19.19)	38(19.10)	
7–8	35(7.45)	18(6.64)	17(8.54)	
Leg_Pain_NRS				<0.001
0–2	19(4.04)	9(3.32)	10(5.03)	
3–4	116(24.68)	47(17.34)	69(34.67)	
5–6	291(61.91)	193(71.22)	98(49.25)	
7–8	44(9.36)	22(8.12)	22(11.06)	
High risk occupation				<0.001
No	148(31.49)	47(17.34)	101(50.75)	
Yes	322(68.51)	224(82.66)	98(49.25)	
Numbness after				0.4124
No	315(67.02)	177(65.31)	138(69.35)	
Yes	155(32.98)	94(34.69)	61(30.65)	
Reduction of lumbago				<0.001
No	339(72.13)	148(54.61)	191(95.98)	
Yes	131(27.87)	123(45.39)	8(4.02)	
Reduction of leg				<0.001
No	349(74.26)	151(55.72)	198(99.50)	
Yes	121(25.74)	120(44.28)	1(0.50)	
JOA improvement				<0.001
No	25(5.32)	25(9.23)	0	
Yes	445(94.68)	246(90.77)	199(100.00)	
ODI difference				<0.001
No	81(17.23)	66(24.35)	15(7.54)	
Yes	389(82.77)	205(75.65)	184(92.46)	
Reoperation				0.0012
No	454(96.60)	255(94.10)	199(100.00)	
Yes	16(3.40)	16(5.90)	0	
Recurrence				<0.001
No	442(94.04)	243(89.67)	199(100.00)	
Yes	28(5.96)	28(10.33)	0	

CTT, Conservative treatment time; WLPT, Waist leg pain time, SLETA, Straight leg elevation test angle of affected limb, DOS, Disturbance of sensation; MS, Muscle strength; Number, Number of salient segments; SSN, Surgical segment number; Segment, Number of operative segments; Collapsa, Collapse of intervertebral space; LS, Lumbar spondylolisthesis; Calcification, Calcification of ligaments hyperplasia of bone; SD, Sagittal diameter; Position, Sagittal disk herniation horizontal position; Location, Transected herniated disk location; Grade, Grading of transected disk herniation; Numbness after, Numbness in the year after surgery; Reduction of lumbago, Reduction of lumbago NRS 1 year after surgery ≧2; Reduction of leg, Reduction of leg pain NRS 1 year after surgery>2; JOA improvement, JOA improvement rate 1 year after surgery ≧ 25; ODI difference, ODI difference 1 year after surgery>20; Proximal lumbar process, Proximal lumbar process within 1 year after surgery; Recurrence, Recurrence occurred within 1 year after surgery.

### Outcome indicators

Cure rate scores for lumbar JOA score at 1 year after TMD surgery were calculated using the same method as before the operation. The cure rate was calculated as follows:


$$\left[\begin{array}{l} \left(\mathrm{post}\;\mathrm{treatment}\;\mathrm{score}-pre\;\mathrm{treatment}\;\mathrm{score}\right)÷\\ \left(\mathrm{full}\;\mathrm{score}\;29-pre\;\mathrm{treatment}\;\mathrm{score}\right)\times 100\% \end{array}\right]$$

This rate reflects the improvement of lumbar spine function before and after treatment, and is utilized to evaluate the clinical efficacy of the intervention. A cure rate of 100% indicates complete recovery, while a cure rate of greater than 60% is considered to be significantly effective. Improvement rates falling within the range of 25–60% are categorized as effective, while those below 25% are classified as ineffective. To process the data, patients with an improvement rate of lumbar JOA score > 60% (significant efficacy or cure) 1 year after TMD were recorded as 1, while patients with an improvement rate of lumbar JOA score ≤ 60% (effective but not significant or ineffective) were recorded as 0.

### Feature engineering

Feature engineering is a process that involves transforming raw data into features that are more suitable for modeling. By doing so, the resulting features are able to capture relevant patterns, thereby improving the predictive accuracy of machine learning and deep learning models on unseen data (15).

In this study, the feature engineering process began by transforming raw data into more suitable features for modeling through data preprocessing and feature selection. Missing values were addressed using mean interpolation (16, 17), and the data were standardized using *Z*-score normalization to ensure uniformity, with all features having a mean of 0 and a standard deviation of 1. Further, before applying the features to eight different predictive algorithms, feature selection was carried out using the Mann–Whitney U test, retaining only those features with *p* values less than 0.05. To reduce redundancy, a Spearman correlation matrix heatmap was used to identify highly correlated features (|ρ| > 0.9), which were eliminated, except for one retained to maintain descriptive power. The final selection utilized LASSO regression with 10-fold cross-validation to identify features with non-zero coefficients essential for modeling.

### Spearman ρ correlation matrix heat map

We conducted a correlation analysis of the data using a Spearman ρ correlation matrix heat map (18). The Spearman correlation matrix heat map is suitable for analyzing data that do not conform to a normal distribution, as well as data that contain categorical variables. It can measure the correlation between any two variables, with a value of +1 indicating a total positive correlation, −1 indicating a total negative correlation, and 0 indicating no correlation. The results of the correlation analysis can be visually represented using a heat map, which uses color to indicate the magnitude of the correlation, making it easier and more intuitive to interpret the results.

### Machine learning and deep learning

We employed a systematic framework based on machine learning and deep learning to construct prognostic models. To this end, we divided the data into a training dataset for developing the predictive model and a test dataset for evaluating the accuracy of the model (19). The data were randomly divided into two groups in a ratio of 70:30, with 70% (*n* = 329) of the samples designated as the training set for developing the predictive model, and 30% (*n* = 141) of the samples designated as the test set for evaluating the accuracy of the model. Once the training set was defined, an optimal model was developed using eight different machine learning algorithms, including Random Forests, Extreme Gradient Boosting, Support Vector Machines, Extra Trees, K-Nearest Neighbors, Logistic Regression, Light Gradient Boosting Machine, and MLP (Artificial Neural Networks) from scikit-learning (version: 0.18) in python.

To optimize the accuracy of the predictive models, a grid search was conducted on the hyperparameters for each of the eight ML algorithms used. A 10-fold cross-validation was employed, whereby the training data set was divided into 10 equally-sized folds, and the model was created using 90% of the data in each fold, with the remaining data used to evaluate the model’s accuracy. The process was repeated 10 times, with each fold being used for one of the 10 training steps (20, 21). The area under the receiver operating characteristic (ROC) curve, also known as area under the curve (AUC), was used as the primary accuracy metric during the grid search (22). The AUC is a performance measure that evaluates the strengths and weaknesses of the learner and is widely used in clinical settings to assess the performance of ML algorithms on test datasets (23). In addition to the AUC, Accuracy, AUC, Sensitivity, Specificity, PPV, NPV, Precision, Recall, and F1 values were also reported to provide a comprehensive picture of the algorithm’s performance (22).

The modeling and prediction process for deep learning is similar to traditional machine learning, with the main difference being that deep learning is end-to-end and can automatically extract high-level features, greatly reducing the reliance on feature engineering in traditional machine learning (7).

### Statistical analysis

Continuous variables were presented as mean ± standard deviation, while categorical variables were presented as frequencies and percentages. Group comparisons for categorical variables were conducted using the chi-square test or Fisher’s exact test, whereas differences between groups for quantitative variables were assessed using the *t*-test or Mann–Whitney U test. Statistical analyses were conducted at a significance level of 0.05 (two-tailed) using Python (version 3.9, http://www.python.org). A two-sided *p* value <0.05 was deemed statistically significant.

## Results

### General

A total of 470 patients meeting the inclusion and exclusion criteria were enrolled in this study. All patients underwent TMD surgery between January 2018 and January 2021 and were followed up for 1 year. In order to develop predictive models, 42 variables were collected, including gender, age, BMI, medical history, and preoperative indicators.

### Correlation matrix heat map

**Figure 1:**
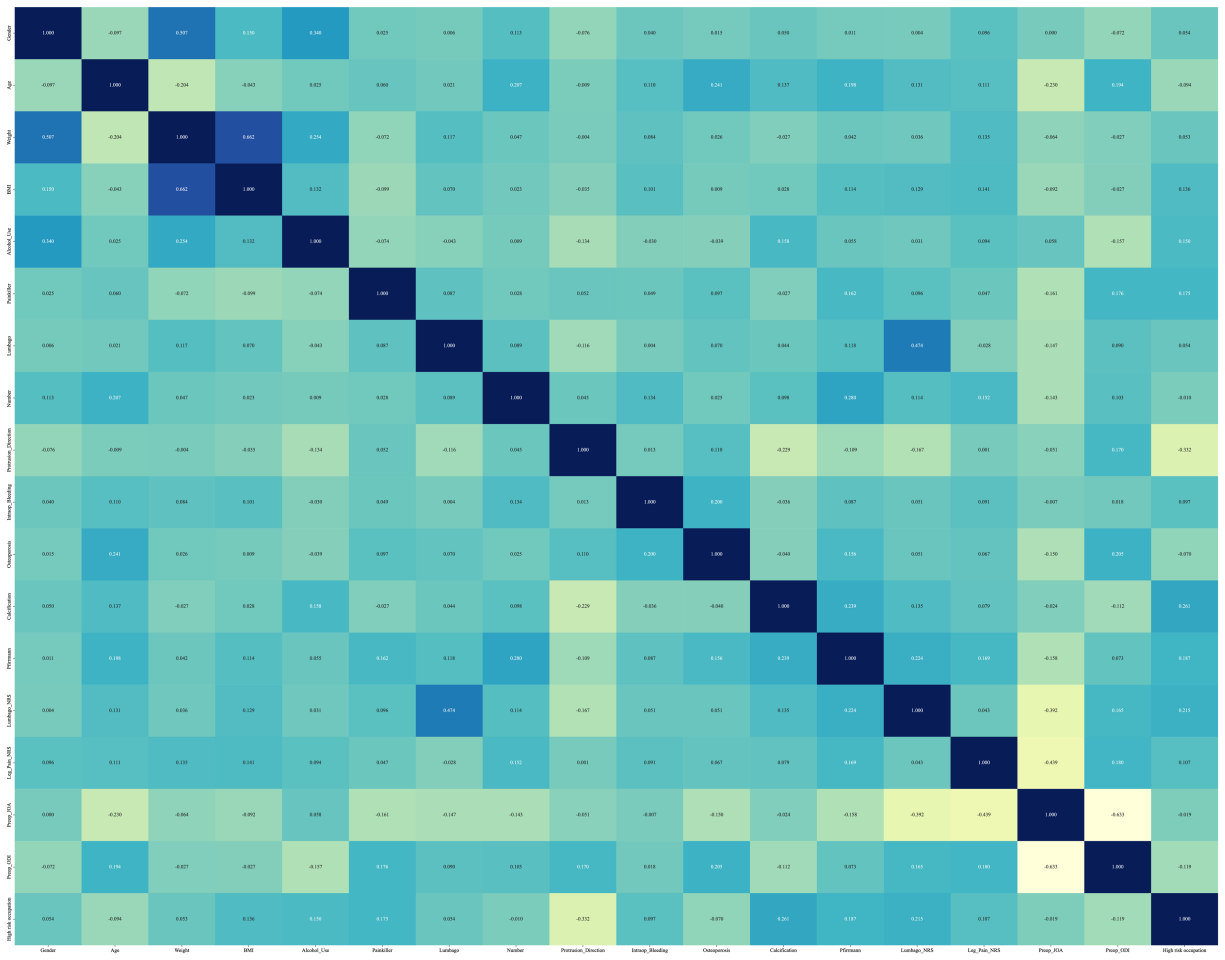
The Spearman ρ correlation matrix heat map used to construct the model independent variables. A large number of highly correlated features are eliminated.

Figure 1 presents the Spearman ρ correlation matrix heatmap, which is utilized to construct the model’s independent variables. This heatmap reveals that there is a medium to strong correlation between several pairs of variables: weight and gender ρ = 0.507, BMI and weight ρ = 0.662, Lumbago-NRS and Lumbago ρ = 0.474, Preop_JOA and leg_pain_NRS ρ = −0.439, and Preop_JOA and Preop_ODI ρ = −0.633. The absolute strength of all other correlations did not exceed 0.40 (│ρ│ ≤ 0.40).

### Machine learning and deep learning

**Figure 2:**
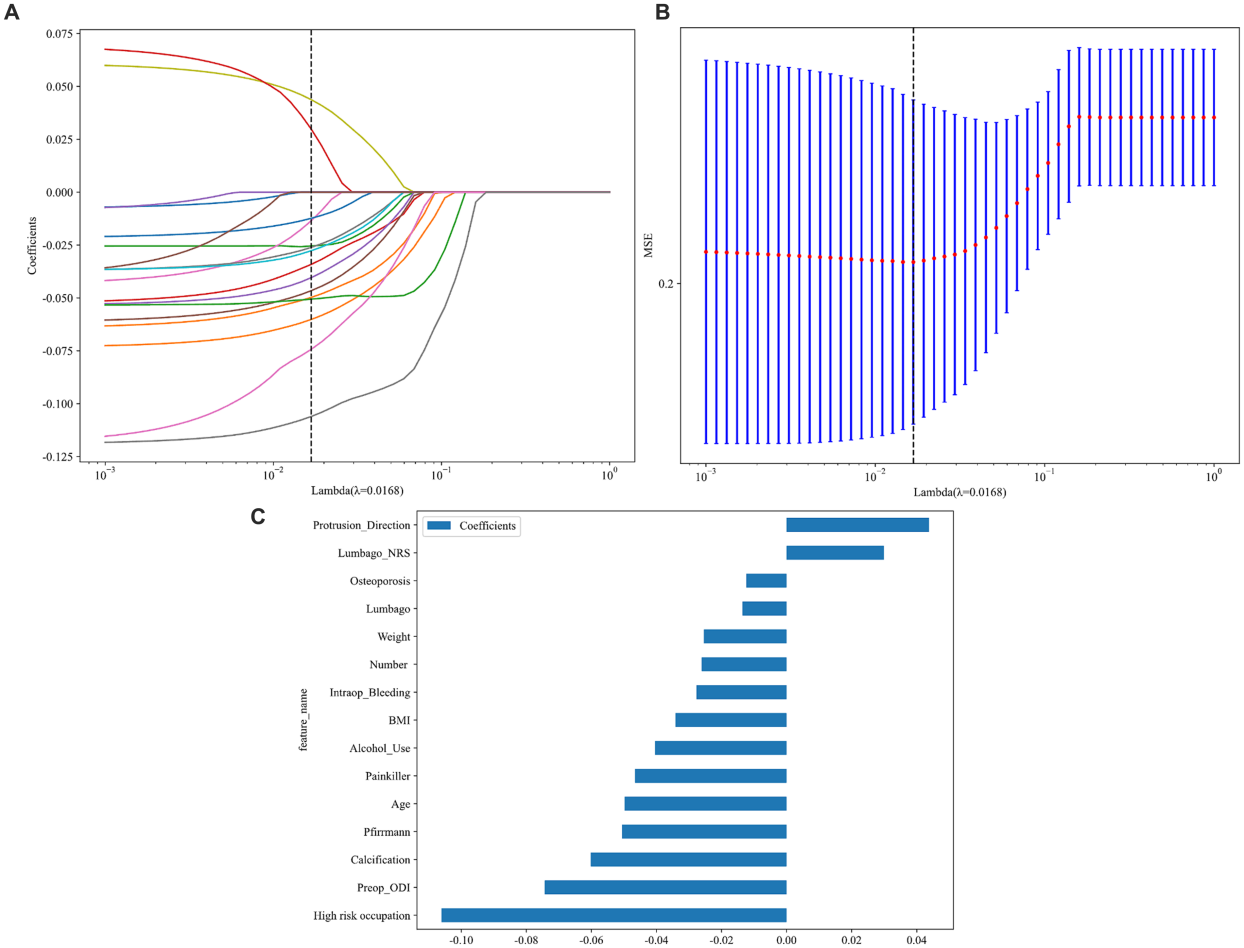
The LASSO and MSE in feature engineering and the 15 variables used to input into eight algorithms. **(A)** The least absolute shrinkage and selection operator (LASSO); **(B)** A 10-fold-validated mean squared error (MSE); **(C)** feature weights: variables-score histogram derived from LASSO-selected features.

After performing data preprocessing and segmenting the dataset into training and test sets, this study employed eight algorithms to develop the predictive model. Finally, 15 variables after Feature Engineering (Figure 2C) were used to input DL and ML algorithm, including high-risk occupation, preop_ODI, calcification, and other 12 variables. Each algorithm was also subjected to a hyperparameter grid search based on a 10-fold cross-validation and after finding the optimal hyperparameters, the models were used to generate predictions.

**Figure 3:**
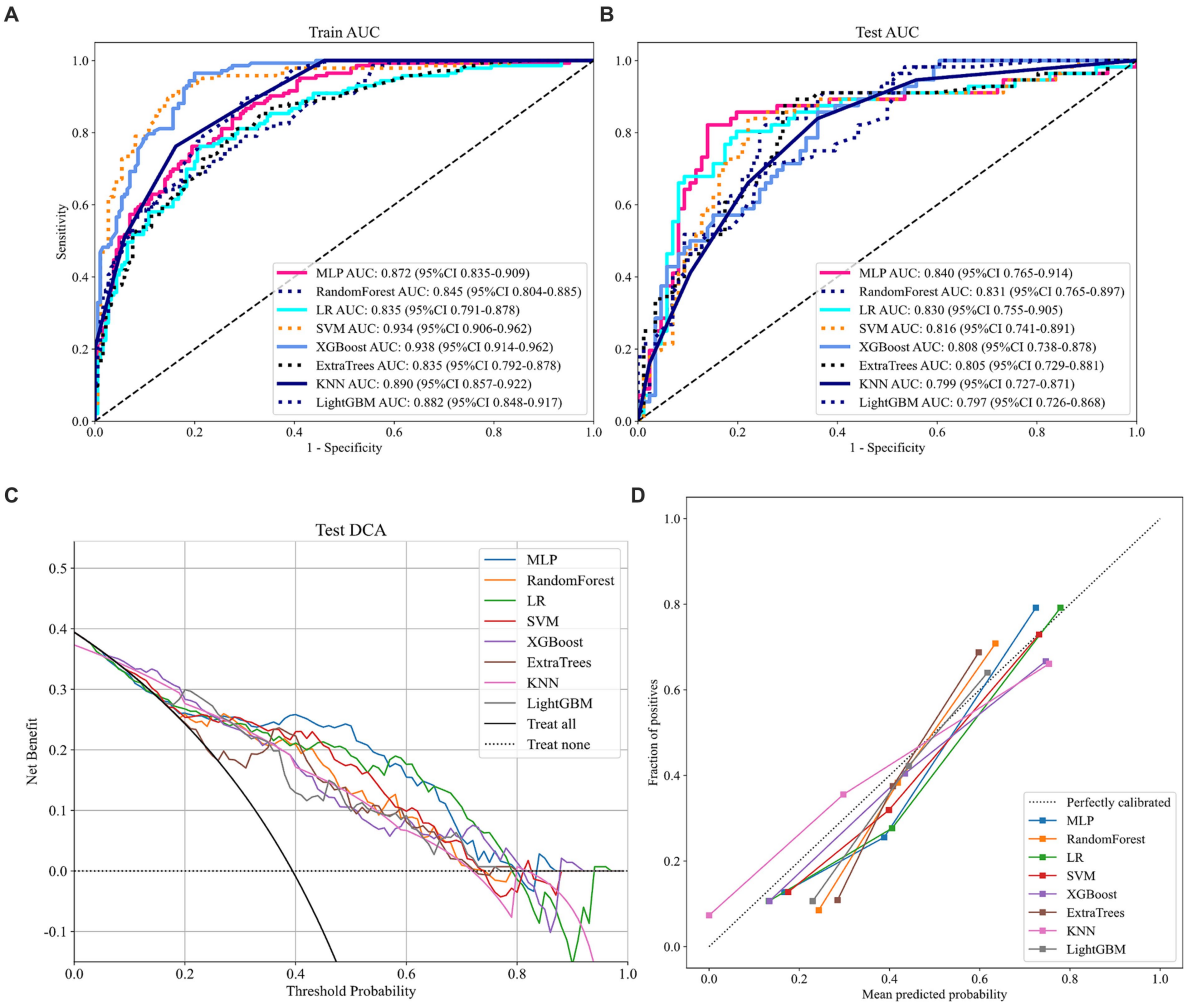
Relevant prediction results of the eight models. **(A)** ROC curve of the train cohort; **(B)** ROC curve of the test cohort; **(C)** DCA curve of the test cohort; and **(D)** Probability calibration curve of the test cohort.

As shown in Figure 2 and Table 2, MLP exhibits the highest AUC values (Train AUC = 0.872; Test AUC = 0.840), also demonstrating superior performance across other metrics such as an Accuracy of 0.8380, Sensitivity of 0.8040, and Specificity of 0.8600 in test cohort (Figures 3A,B). Additionally, Figure 3C illustrates the superior clinical decision-making capability of MLP (represented by the blue curve) at thresholds greater than 40% (DCA), where it demonstrates a higher net benefit compared to other machine learning algorithms. The Probability Calibration Curve also supports our decision-making process (Figure 3D). Performance comparisons of each model are detailed in Table 2.

**Table 2:** The performance of each model evaluated by accuracy, AUC, sensitivity, specificity, PPV, NPV, Precision, Recall, and F1.

Models	Cohort	Accuracy	AUC	95% CI	Sensitivity	Specificity	PPV	NPV	Precision	Recall	F1
MLP	Train	0.7840	0.8720	0.8347–0.9088	0.7550	0.8050	0.7500	0.8100	0.7500	0.7550	0.7530
RandomForest	Train	0.7620	0.8450	0.8044–0.8854	0.6290	0.8650	0.7830	0.7510	0.7830	0.6290	0.6980
LR	Train	0.7740	0.8350	0.7910–0.8783	0.7550	0.7890	0.7350	0.8070	0.7350	0.7550	0.7450
SVM	Train	0.8690	0.9340	0.9064–0.9615	0.9160	0.8320	0.8090	0.9280	0.8090	0.9160	0.8590
XGBoost	Train	0.8690	0.9380	0.9143–0.9619	0.9580	0.8000	0.7870	0.9610	0.7870	0.9580	0.8640
ExtraTrees	Train	0.7560	0.8350	0.7920–0.8779	0.7970	0.7240	0.6910	0.8220	0.6910	0.7970	0.7400
KNN	Train	0.7530	0.8900	0.8573–0.9223	0.5100	0.9410	0.8690	0.7130	0.8690	0.5100	0.6430
LightGBM	Train	0.7800	0.8820	0.8476–0.9166	0.8740	0.7080	0.6980	0.8790	0.6980	0.8740	0.7760
MLP	Test	0.8380	0.8400	0.7651–0.9143	0.8040	0.8600	0.7890	0.8710	0.7890	0.8040	0.7960
RandomForest	Test	0.7750	0.8310	0.7649–0.8974	0.8040	0.7560	0.6820	0.8550	0.6820	0.8040	0.7380
LR	Test	0.7960	0.8300	0.7550–0.9049	0.7860	0.8020	0.7210	0.8520	0.7210	0.7860	0.7520
SVM	Test	0.7960	0.8160	0.7409–0.8912	0.8210	0.7790	0.7080	0.8700	0.7080	0.8210	0.7600
XGBoost	Test	0.7180	0.8080	0.7381–0.8776	0.8390	0.6400	0.6030	0.8590	0.6030	0.8390	0.7010
ExtraTrees	Test	0.7610	0.8050	0.7294–0.8808	0.8390	0.7090	0.6530	0.8710	0.6530	0.8390	0.7340
KNN	Test	0.7320	0.7990	0.7266–0.8706	0.6610	0.7790	0.6610	0.7790	0.6610	0.6610	0.6610
LightGBM	Test	0.7180	0.7970	0.7257–0.8681	0.6790	0.7440	0.6330	0.7800	0.6330	0.6790	0.6550

## Discussion

In the field of surgical treatment for disk herniation, there have been numerous studies investigating the efficacy of different surgical approaches. Specifically, research has focused on the differences in treatment outcomes between TMD and other approaches, such as open microdiscectomy (OMD). Studies have demonstrated that TMD and OMD yield comparable treatment outcomes, but TMD has a significant advantage in reducing intraoperative bleeding (24). Additionally, research has shown that TMD and conventional microdiscectomy (CMD) produce similar outcomes 1 year after surgery, with TMD not having any advantage in preventing reoperation or dural tears (25). However, limited discussion has been dedicated to patient recovery 1 year after TMD. This study provides a novel approach to addressing the lack of research in this area by implementing machine learning and deep learning techniques to develop predictive models for patient recovery 1 year after TMD.

A limited amount of central data can also be used for deep learning predictive analysis and may be useful for clinical decision making (26). Its comparison of logistic regression models with deep learning models shows the superiority of deep learning performance. Our prediction results demonstrate the advantages of MLP models, especially in terms of AUC values. Of course, close results were obtained for LR, RF, etc., which may be related to the small amount of data, coming from a single clinical study center.

Logistic regression without regularization may be criticized for underfitting, but L2-regularized logistic regression effectively mitigates the risk of overfitting by incorporating a regularization factor or penalty factor, denoted as λ, which multiplies the sum of the squares of all parameters. This reduces the impact of insignificant parameters on the predictive outcome.

Wang et al. (27) previously utilized a stepwise logistic analysis to filter parameters and select the optimal independent variable based on the minimum Akaike information criterion (AIC) as input for their machine-learning algorithm. Although this study did not utilize this particular machine-learning algorithm, we standardized our data through *Z*-score normalization. This will reduce the influence of outliers on the model fit. While the correlation matrix heat map is a valuable tool, we acknowledge that the screening process could lead to the exclusion of crucial independent variables. Moreover, the selection of the step probability directly influences the screening outcome. If the step probability is set too low, a substantial number of independent variables may be omitted. On the other hand, increasing the step probability could still result in the loss of important independent variables due to the limited amount of available data, thereby rendering the method meaningless.

Prognostic models offer clinicians an effective means of conveying quantitative risk predictions to patients, thus mitigating information asymmetry to some extent. Accurate determination of surgical indications using such models would enable clinicians to focus their attention on tasks that cannot be automated. Unfortunately, achieving this goal is currently challenging. The primary obstacle lies in the absence of external model validation, which is necessary to ensure its generalizability to other datasets. The solution may involve conducting multicenter studies to improve the predictive accuracy and generalizability of prognostic models.

In addition to the limitations of data volume, this study has several noteworthy shortcomings. (1) The retrospective nature of the study may have introduced selection bias, undermining the generalizability of the findings. (2) Despite our attempts to collect data on a wide range of variables that may impact the improvement rate of JOA 1 year after surgery, there is a possibility that important variables were overlooked. (3) Due to hardware constraints and the need for machine learning expertise, large-scale generalization of our findings is currently difficult to achieve. (4) The sample size in this study is relatively small, and as a single-center study, additional more data and more centers in the future might enhance our results. Finally, in this study, we used retrospective data for predictive modeling, and in the future, we need to add prospective data for further analysis, which will enhance our clinical evidence.

## Data availability statement

The raw data supporting the conclusions of this article will be made available by the authors, without undue reservation.

## Ethics statement

The study was conducted according to the guidelines of the Declaration of Helsinki, and was approved by Institutional Review Board (approval no. 2022KY026). The studies were conducted in accordance with the local legislation and institutional requirements. Written informed consent for participation was not required from the participants or the participants’ legal guardians/next of kin in accordance with the national legislation and institutional requirements.

## Author contributions

YC: Methodology, Writing – original draft. FL: Methodology, Writing – original draft. KW: Writing – review & editing. FC: Formal Analysis, Visualization, Writing – review & editing. RuxW: Writing – original draft. ML: Data curation, Writing – original draft. CC: Project administration, Supervision, Writing – review & editing. RuiW: Project administration, Supervision, Writing – review & editing.
