# Supplementary material for: Development of a predictive model for 1-year postoperative recovery in patients with lumbar disk herniation based on deep learning and machine learning
Source: Front Neurol. 2024 Jun 11;15:1255780. doi: 10.3389/fneur.2024.1255780 (PMC11197993; doi:10.3389/fneur.2024.1255780)
Supplement: Supplementary file 1 [file Data_Sheet_1.docx]

Basic information

Age, gender, height, weight, body mass index (BMI), high-risk occupation (an occupation that requires long-term sedentary standing or heavy physical activity), and family history (a first-degree relative with LDH).

Medical history examination

History of lumbar trauma, duration of disease, preoperative conservative treatment time, preoperative pain medication use time, low back pain, underlying diseases (hypertension and diabetes), smoking history, alcoholism, preoperative physical examination (straight leg raising test) angle, sensory disturbance, muscle strength grading of the affected limb, and Barthel's scale.

Preoperative inspection indicators

Serum creatine kinase (CK).

Preoperative examination indicators

(1) Degeneration of the lumbar spine: ligament calcification, osteoporosis, lumbar spondylolisthesis, lumbar intervertebral disc space collapse, lumbar spinal canal sagittal meridian, and Modic type changes in the endplate and subendplate (Figure 1).

(2) Related conditions of lumbar intervertebral disc herniation: the number of protruding segments, the protruding position of the responsible segment, the sagittal division of the responsible segment, transverse division of the responsible segment and the degree of herniation according to the Michigan State University (MSU, MSU) classification grade, and Pfirrmann grade for lumbar intervertebral disc degeneration of the responsible segment.

Preoperative scoring

(1) The American Society of Anesthesiologists (ASA) score: before anesthesia, the patient was divided into 5 grades according to the patient's physical condition and surgical risk (grade 1 means no other systemic diseases except local lesions, grade 2 indicates mild or moderate systemic disease, grade 3 indicates that the patient has any serious systemic disease but has not lost the work ability, grade 4 indicates that the patient has a serious life-threatening systemic disease and has lost the work ability, and grade 5 indicates that the patient is in a critical condition requiring emergency surgery).

(2) Oswestry Disability Index (ODI) score: a total score of 10 for the pain level, self-care ability of daily living, carrying objects, walking, sitting, standing, sleep, sex life, social activities, and travel was assessed by a questionnaire. Each aspect has 1 question and 6 options, corresponding to 0 to 5 points, respectively. The ODI score was calculated as the percentage of the total score obtained by the subjects in the total score of the highest score of the evaluated item. The higher the score, the more severe the dysfunction.

(3) Numerical rating scale (NRS) score for preoperative low back pain and leg pain: a score of 0-10 indicates the degree of pain; the higher the value, the higher the degree of pain.

(4) The Japanese Orthopedic Association (JOA) evaluation treatment score for the lumbar spine before surgery: including subjective symptoms (such as the degree of low back pain, numbness or pain in the lower extremity, and walking ability), clinical signs (such as straight leg lift) High angle, sensory disturbance, muscle strength 3 items), the degree of limitation of daily activities (including bed rest, standing, washing, bending, sitting, lifting, and walking; 7 items) and bladder function in a total of 4 aspects, each with 3- . The four choices correspond to their respective scores, with a total score. The lower the score, the more obvious the dysfunction.

Surgery-related indicators

Surgical segments, number of operative segments, operative time, and intraoperative blood loss were considered as the surgery-related indicators.
